# Robust Resistive Switching Constancy and Quantum Conductance in High-*k* Dielectric-Based Memristor for Neuromorphic Engineering

**DOI:** 10.1186/s11671-022-03699-z

**Published:** 2022-06-24

**Authors:** Muhammad Ismail, Chandreswar Mahata, Myounggon Kang, Sungjun Kim

**Affiliations:** 1 Division of Electronics and Electrical Engineering, Dongguk University, Seoul, 04620 Republic of Korea; 2 Department of Electronics Engineering, Korea National University of Transportation, Chungju-si, 27469 Republic of Korea

**Keywords:** Quantum conductance, Neuromorphic synapses, High switching stability, High ON/OFF ratio, HfO_2_ switching layer, Interface engineering

## Abstract

For neuromorphic computing and high-density data storage memory, memristive devices have recently gained a lot of interest. So far, memristive devices have suffered from switching parameter instability, such as distortions in resistance values of low- and high-resistance states (LRSs and HRSs), dispersion in working voltage (set and reset voltages), and a small ratio of high and low resistance, among other issues. In this context, interface engineering is a critical technique for addressing the variation issues that obstruct the use of memristive devices. Herein, we engineered a high band gap, low Gibbs free energy Al_2_O_3_ interlayer between the HfO_2_ switching layer and the tantalum oxy-nitride electrode (TaN) bottom electrode to operate as an oxygen reservoir, increasing the resistance ratio between HRS and LRS and enabling multilayer data storage. The Pt/HfO_2_/Al_2_O_3_/TaN memristive device demonstrates analog bipolar resistive switching behavior with a potential ratio of HRS and LRS of > 10^5^ and the ability to store multi-level data with consistent retention and uniformity. On set and reset voltages, statistical analysis is used; the mean values (*µ*) of set and reset voltages are determined to be − 2.7 V and + 1.9 V, respectively. There is a repeatable durability over DC 1000 cycles, 10^5^ AC cycles, and a retention time of 10^4^ s at room temperature. Quantum conductance was obtained by increasing the reset voltage with step of 0.005 V with delay time of 0.1 s. Memristive device has also displayed synaptic properties like as potentiation/depression and paired-pulse facilitation (PPF). Results show that engineering of interlayer is an effective approach to improve the uniformity, ratio of high and low resistance, and multiple conductance quantization states and paves the way for research into neuromorphic synapses.

**Supplementary Information:**

The online version contains supplementary material available at 10.1186/s11671-022-03699-z.

## Introduction

Because of their simple structure, high-density integration, low power consumption, and fast operation, memristor devices are gaining a lot of interest for memory, logic, neural networks, and sensing applications [1, 2]. In particular, two-terminal device may modify its resistance in response to electrical stimulation of voltage pulses and stores data [3]. Furthermore, memristive devices offer internal computing capabilities and enable a novel computing paradigm, allowing calculation results to be generated and stored on-site without the need for data movement operations, avoiding von Neumann's bottleneck [4]. In oxide-based memristive devices, resistive switching is attributed to the formation/rupture of oxygen-related defects/vacancies or cation migration made conducting filaments [5–7]. Further to explain the working principle of the memristive device with an active electrode, electrochemical reaction and cation migration are the most recognized mechanism, which is similar to the valance change mechanism [8]. However, memristive devices based on transition metal oxides, such as HfO_2_, Al_2_O_3_, Ta_2_O_5_, and ZrO_2_ along with others, suffer from non-uniformity in resistive switching parameters, such as instability in resistance values of low- and high-resistance states (LRSs and HRSs), and dispersion in set and reset voltages, which obstruct their commercial applications [9–19]. To address these issues, inserting an interlayer [20, 21], which can function as an oxygen reservoir and possibly controls oxygen ions concentration in the original and inserted layers, thereby improving resistive switching parameters [22], is conducive in enhancing resistive switching performance. In transition metal oxides-based memristive devices, oxygen ions movement and their distribution usually play a substantial role in resistive switching behavior, which is now widely accepted [23, 24]. In a previous work, we have shown that by inserting a thin layer (such as ZTO, CeO_2_, TiO_2_, and HfO_2_) to remove resistive switching parameters instability and their dispersion is a practical and effective method [20, 21, 25–27].

It is worth noting that, in recent years, resistance switching devices are being utilized in advanced brain-inspired applications [28, 29]. Such devices are used to replicate biological processes and so might be the game changer in breaking the von Neumann structural bottleneck. Electrical impulses are able to modify the current flowing through such resistive changing devices; this behavior is analogous to the functions of biological synapses [30–33]. Since in both pre- and post-neurons, a biological synapse changes its weight (equivalent to the conductance of a memristor) by discharging Ca^2+^ or Na^+^ ions [34, 35]. The potentiation and depression are important mechanisms in a biological nervous system, which indicates a deep-rooted transformation in the connection strengths between neurons. According to the interval between presynaptic and postsynaptic action potentials or spikes, the phenomenon of synaptic weight modification is known as spike-timing-dependent plasticity [36]. Due to scalability, low power operation, nonvolatile features, and small on-chip area, memristors are good candidates for artificial synaptic devices to mimic potentiation, depression, and spike-timing-dependent plasticity behaviors [3, 37, 38]. The conductivity of resistive switching devices might be gradually modified by adjusting the input stimuli, like the resistive switching process, in view of ionic migration and charge carrier trapping/detrapping. [39–41] The resistive switching devices could be used to simulate the functions of artificial synapses because their features are identical to those of biological synapses. The nanoscale kinetics for the conduction channel development could explain the progressive shift in conduction under the influence of an electric field [6]. Despite such limitations, the development of conductive routes and their dynamic visualization of neuromorphic behavior have still remained the fundamental and unsolved puzzle that necessitates a significant technological advancement.

Furthermore, reducing the conductive filaments to the atomic scale of quantum point contact permits memristive ballistic electron transport in analog domains without scattering and quantized conductance characteristics [42–44]. It not only dramatically enhances the data storage capability of gadgets, but it also allows neuromorphic systems to analyze information more efficiently. Multiple conductance states of memristor devices should, in general, be realized in as simple a manner as possible for practical use [45–47]. Nonetheless, the most investigations to-date have relied on sophisticated programming approaches to obtain multi-conductance characteristics, with the addition of varying current compliances and voltages putting a strain on the overall circuit design [30, 48, 49]. In resistive switching memristors, improvements are desperately needed not only to simplify the operational philosophy but also to provide dependable and analog-type conductance quantization behavior.

In this paper, we have fabricated Pt/HfO_2_/Al_2_O_3_/TaN bilayer structure through atomic laser deposition (ALD) to explore the multilevel conductance quantization for neuromorphic synapses. Uniform resistance distributions, large ON/OFF ratio (> 10^5^), low working voltage (− 2.9/+ 1.7 V), fast speed (1.2/2.0 µs), consistent DC endurance (1000 cycles), AC endurance 10^5^ cycles, long-term retention (10^4^ s) features, and multilevel quantized conductance states are among the resistive switching characteristics of the memristive device. Interestingly, we have also observed synaptic behavior related to neural learning functions, such as potentiation, depression, and paired-pulse facilitation. The oxygen vacancy-based conducting filamentary schematic model has been proposed to illustrate the resistive switching mechanism. Results show that Pt/HfO_2_/Al_2_O_3_/TaN memristive devices have sufficient potential for their practical applications as high data storage memory and electronic synapses.

## Experimental

By creating the structure of Pt/HfO_2_/TaN with Al_2_O_3_ interlayer, two-terminal memristive devices based on HfO_2_ film were developed in this experiment. First, a ~ 2 nm Al_2_O_3_ interlayer was deposited on a TaN/Si substrate through atomic layer deposition (ALD) for 23 cycles utilizing trimethylaluminum (TMA) as the Al source and H_2_O as the oxidizing agent (as O_2_ source). Then, using ALD, a 5-nm HfO_2_ switching layer was deposited using Tetrakis dimethylamino hafnium (TDMAHf) and H_2_O as the Hf and O precursors, respectively, with one oxide cycle consisting of 0.5 s Hf metal source injection, 6 s N_2_ purging, 0.5 s H_2_O injection, and 20 s N_2_ purging. At 260 °C, the TEMAH was evaporated. Pure N_2_ (99.999 percent purity) was employed as a carrier gas and purge gas. Finally, a 100-nm-thick Pt top electrode with a diameter of 100 µm was deposited with a circular metal shadow mask by using e-beam evaporation technique to obtain the Pt/HfO_2_/Al_2_O_3_/TaN memristive device. Electrical and pulse measurements were taken with a Keithley SCS 4200A parameter analyzer system on a probe station in ambient conditions. Based on the current going from top to bottom electrode, the bias was determined to be positive in this experiment. The cross-sectional view and film thickness of the HfO_2_/Al_2_O_3_/TaN structure were confirmed using a high-resolution transmission electron microscope (HRTEM, JEOL/CEOS, JEM-2100F, Cs corrector). The chemical composition and bonding states of the HfO_2_/Al_2_O_3_/TaN structure were further studied using X-ray photoelectron spectroscopy (XPS, Thermo Fisher K-Alpha) using a monochromatic Al K source (*hv* = 1486.6 eV) for photoelectrons excitation. The charge effect was calibrated by setting the C 1s photoemission at 284.6 eV. The XPS depth profile of HfO_2_/Al_2_O_3_ on TaN-coated Si was obtained using Ar ion etching.

## Results and discussion

### Memristor design and structural characterization

**Fig. 1:**
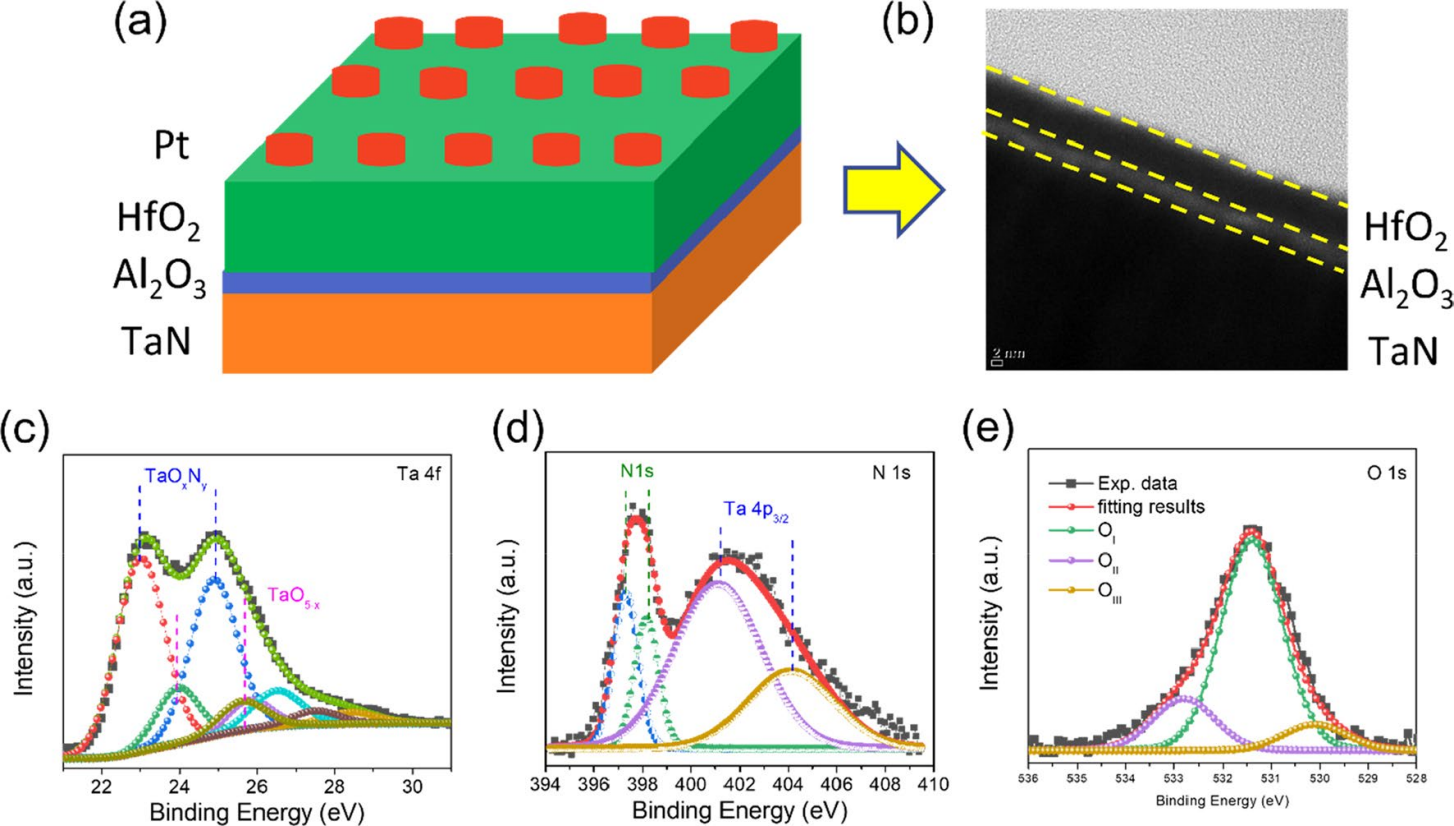
**a** Schematic diagram of Pt/HfO_2_/Al_2_O_3_/TaN memristive device. **b** Cross-sectional TEM image of HfO_2_/Al_2_O_3_/TaN structure. XPS analyses of TaO_x_N_y_ interfacial layer; **c** Ta 4f, **d** N 1s, **e** O 1s

Figure 1 reveals the schematic configuration and structural characterization of Pt/HfO_2_/Al_2_O_3_/TaN memristive device. The schematic construction of a Pt/HfO_2_/Al_2_O_3_/TaN memristive device is shown in Fig. 1a. The HRTEM was used to obtain a cross-sectional view of the HfO_2_/Al_2_O_3_ bilayer film to confirm the thickness of each layer and the formation of an interfacial layer at the Al_2_O_3_/TaN bottom interface. Figure 1b shows a HRTEM image of the HfO_2_/Al_2_O_3_/TaN structure. HfO_2_ and Al_2_O_3_ films have thicknesses of 5 and 2 nm, respectively. We also did XPS investigations on HfO_2_/Al_2_O_3_/TaN to gain a better understanding of the switching process in the bilayer structure of HfO_2_/Al_2_O_3_ on TaN-coated Si. XPS spectra were fitted with 5% Gaussian–Lorentzian functions after smart-type background subtraction. In the supplementary information, the narrow-scan XPS spectra of Al 2p and Hf 4f are given in Additional file 1: Fig. S1. The Hf 4f peaks of 18.4 eV and 20.05 eV, which correspond to the Hf 4f_7/2_ and Hf 4f_5/2_ doublets with a 1.65 eV spin–orbit splitting, are consistent with previously published values [50, 51]. Another low-intensity doublet was fitted, corresponding to Hf–O of a non-stoichiometric suboxide bond due to Hf_x_ + 4f_7/2_ and Hf_x_ + 4f_5/2_ peaks (× 4) at 17.15 and 18.75 eV, respectively [52]. Two Gaussian–Lorentzian line form peaks are also present in Al 2p spectra. At 75.5 and 74.3 eV, these Al 2p peaks can be found. The first peak is attributed to Al–O bonding [53], while the second peak is attributed to Ta–Al–O bonding, which could indicate the decomposition of the Al_2_O_3_ interlayer, releasing oxygen ions that can react with TaN to form the TaO_x_N_y_ interfacial layer, which is made possible by the oxidization of the TaN electrode during the Al_2_O_3_ layer deposition [54].

The presence of TaO_x_ at the Al_2_O_3_/TaN interface appears to be responsible for the Ta 4f doublet at 24.1 eV and 26.0 eV, as shown in Fig. 1c. At 23.5 eV, another doublet with a lower binding energy and a higher intensity is observed, indicating the production of TaO_x_N_y_ at the Al_2_O_3_/TaN interface [55]. Multiple peaks in Fig. 1d fit the overlap of N 1 s and Ta 4p_3/2_. The TaN electrode has produced two N 1 s peaks at 397.1 eV and 398.1 eV, which represent differing ratios of N bound to Ta [56]. Peaks associated with Ta-N and TaOxNy have generated with binding energies of 401. 2 eV and 404.1 eV, respectively, from core-level Ta 4p_3/2_. As a result of these XPS findings, it can be deduced that a thin interfacial layer of TaO_x_N_y_ has developed at the Al_2_O_3_/TaN contact. This interfacial layer is predicted to play a key role in increasing the memristive device's switching performance. Figure 1e shows the O1s core-level spectrum for the TaO_x_N_y_ interfacial layer. Three separate peaks may be seen in this spectrum. The strong peak at 531.4 eV (O_I_) corresponds to oxygen in the TaO_x_N_y_ layer, whereas the higher and lower binding energy peaks at 530.1 eV (O_II_) and 532.8 eV (O_III_), respectively, are attributed to hydroxyl and carbonate groups raised from atmospheric exposure due to the TaN metal electrode's high oxygen affinity.

### Electrical Characteristics

**Fig. 2:**
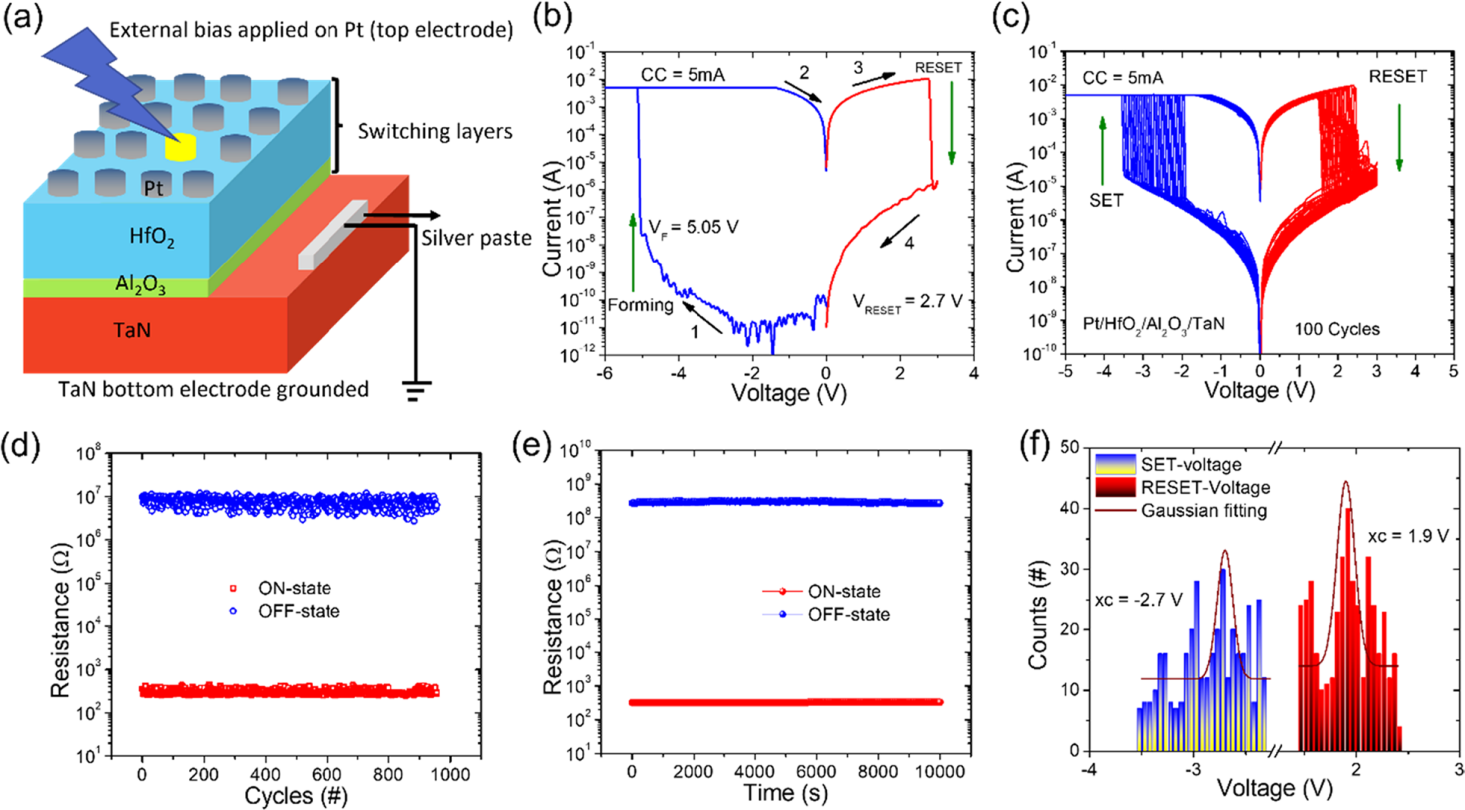
**a** Schematic diagram of Pt/HfO_2_/Al_2_O_3_/TaN memristive device in electrical measurement configuration, which shows that the external voltage is applied to the Pt top electrode with the TaN bottom electrode electrically grounded. **b** I–V curves of an electroforming process (blue) and reset process (red). The arrows and numbers represent voltage sweep directions and sequences, respectively. **c** Typical bipolar I–V characteristics of 100 consecutive cycles, where green arrows indicate the set and reset directions. **d** DC endurance test results over 1000 cycles; the values of ON-state and OFF-state resistances were read at ± 0.2 V under ambient conditions. **e** Data retention characteristics of ON state and OFF state under a constant voltage stress of 0.2 V for 10^4^ s at room temperature. **f** Statistical distribution analyses of set and reset voltages for the 100 switching cycles

Figure 2a displays the schematic diagram of Pt/HfO_2_/Al_2_O_3_/TaN memristive device used for the electrical measurements performed by Keithley 4200-SCS parameter analyzer. The TaN electrode is used to define the bias polarity. The I–V characteristics of the Pt/ HfO_2_/Al_2_O_3_/TaN memristive device are shown in Fig. 2b. The high-resistance state (HRS) of the pristine device is the virgin condition, with a resistance of > 10^10^ at 0.2 V read voltage. An electrical forming technique with high voltage is required to induce reproducible resistive switching behavior in the virgin Pt/ HfO_2_/Al_2_O_3_/TaN memristive device. At 5.05 V of applied negative voltage to the Pt electrode with a 5-mA compliance current, memristive device current abruptly increases, suggesting that the memristive device transitions from the initial-resistance state (IRS) to the low-resistance state (LRS), as illustrated in Fig. 2b. The reset process occurs at a positive bias of about + 2.7 V, where the memristive device switches from LRS to HRS. In order to investigate the average electroforming voltage of the memristive devices, electroforming process was performed on twelve randomly selected memristive cells. Additional file 1: Fig. S2(a) shows the box chart of electroforming voltage of twelve memristive cells, which represent the distribution of formation sites in the conductive filaments and are related to the operating voltage of memristive device. Average electroforming voltage was calculated and is to be − 4.95 as shown in Additional file 1: Fig. S2(b). Furthermore, to examine the electrical homogeneity for high-density memory applications, stability and reproducibility during set and reset operations are of utmost importance. Figure 2c shows the typical I–V curves of 100 continuous set and reset cycles on the Pt/HfO_2_/Al_2_O_3_/TaN memristive device. In the set process under negative bias, only a sudden increase in current is noted, while reset transitions in the positive voltage sweep involve either abrupt (single state) or multi-state transformations. These set and reset processes could only be possible for the opposite bias polarities indicating that the memristive device displays the bipolar resistive switching effect.

Furthermore, one of the most significant properties for nonvolatile memory applications is direct current (DC) cycling endurance. The Pt/HfO_2_/Al_2_O_3_/TaN memristive device has such endurance properties, as shown in Fig. 2d. Switching qualities that are steady and repeatable have been established. At room temperature, the sweeping voltage was applied from 0 to 5 V for set and 0 to 3 V for reset, with a reading voltage of 0.2 V. This endurance test illustrates an excellent durability during continuous 1000 switching cycling. The ON/OFF ratio between HRS and LRS remains around 10^5^ during direct DC switching operation. The data retention properties of the Pt/HfO_2_/Al_2_O_3_/TaN memristive device were further confirmed by monitoring the time-dependent evolution of resistance values in both the LRS and HRS at room temperature. After applying negative (− 5 V) and positive (+ 3 V) bias voltages for a short time, the ON-state and OFF-state resistances were read out. With a read voltage of 0.2 V, the resistance magnitudes were recorded every 10 s. Both LRS and HRS, as shown in Fig. 2e, were constant for 10^4^ s with no discernible decline. After removing the power supply for more than 104 s, the memristive device remains in the ON/OFF state, indicating that it is nonvolatile. The statistical distribution studies of the set and reset voltages of the Pt/HfO_2_/Al_2_O_3_/TaN memristive device are shown in Fig. 1f. Set and reset voltages are found to have typical values of − 2.7 V and 1.9 V, respectively.

The memristive devices performance and memory window could thus be improved by a bilayer or multilayer design. The memristive device with the Pt/HfO_2_/Al_2_O_3_/TaN structure has better time switching stability and reproducibility. As a result, the memristive devices switching stability and consistency were investigated further. Over 120 switching cycles, the dependability and reproducibility of nine randomly selected memristive devices were validated. The typical I–V characteristics of the nine memristive devices are shown in Additional file 1: Fig. S3 (a–i). In addition, as shown in Additional file 1: Fig. S4 (a–i), an endurance test was undertaken. Each memristive device showed consistent switching behavior with no discernible deterioration between the ON and OFF states. The memristive device high ON/OFF resistance ratio (10^5^) and switching characteristics make it ideal for use as electronic synapses.

### Multilevel Data Storage Capability by Controlling Current Compliance

**Fig. 3:**
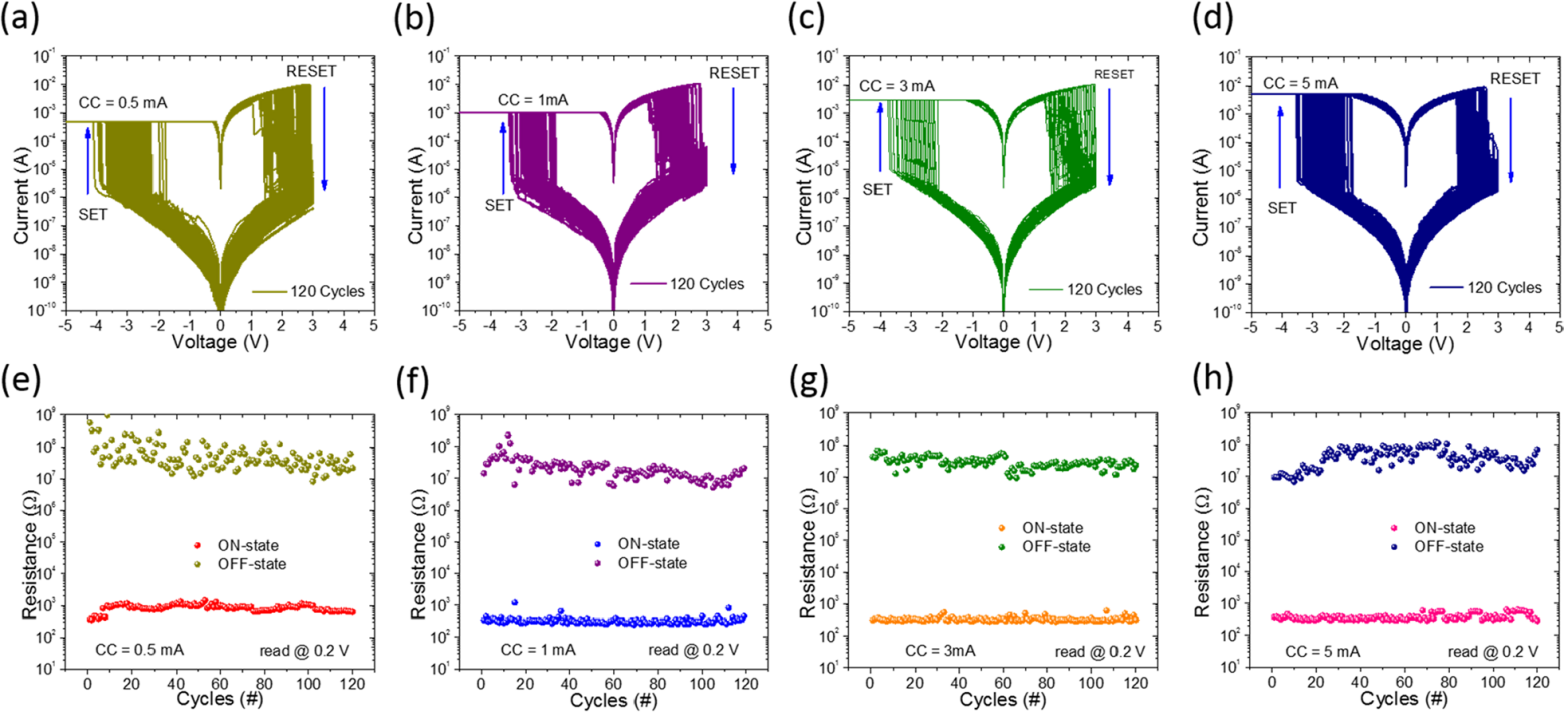
**a**–**d** Typical bipolar I–V curves, and **e**–**h** endurance performance of Pt/HfO_2_/Al_2_O_3_/TaN memristive devices at different current compliances of 0.5 mA, 1 mA, 3 mA, and 5 mA

The literature reports [17, 21, 31] that compliance current is a key factor to influence the resistance distributions during switching operation. During testing operations of memristive devices, a current compliance is usually applied to protect the devices from hard breakdown. In addition, current compliance can greatly affect the conductive filament size in demonstrating the multilevel storage capability [19, 57]. That is why, in the present study, different current compliance magnitudes were applied as varied from 0.5 to 5.0 mA, which are capable to modulate the conductive filament size leading to multilevel data storage capability. Figure 3a–d shows the typical bipolar I–V characteristics of 120 cycles. During measurements, set and reset processes were adjusted with respective voltage sweep of − 5 V and + 3 V under different current compliances (such as 0.5 mA, 1 mA, 3 mA, and 5 mA). Moreover, the reset process was kept free from current compliance. Endurance performance under different current compliances is shown in Fig. 3e–g. Note that current level during ON state under different current compliances (i.e., 0.5 mA, 1 mA, 3 mA, and 5 mA) was almost similar. It means that thicker filaments are formed during set process, so that higher reset voltages are required to rupture thicker conductive filaments. In addition, I–V characteristics were examined by randomly selected memristive cells under varying current compliance (1 mA, 3 mA, and 5 mA) to further corroborate the influence on the ON state of the resistance, as shown in Additional file 1: Fig. S5 (a-e). Only switching cycling uniformity is enhanced by increasing current compliance, almost in the same way as single cell memristive properties are improved by raising current compliance. The schematic representation of a switching mechanism with various current compliances is shown in Additional file 1: Fig. S6. The size (diameter) of conductive filaments does not increase even though additional oxygen vacancies are accumulated under different current compliances, according to the filamentary model. We conclude that memristive devices have capability to operate at each current compliance in almost same manner for its nonvolatile memory applications. All these results indicate that current compliance in the present case does not significantly influence the ON/OFF resistance ratio suggesting that the bilayer memory device is locally conductive and obeys the model of conducting filaments.

### Multilevel Data Storage Capability by Adjusting V_RESET__-stop_

**Fig. 4:**
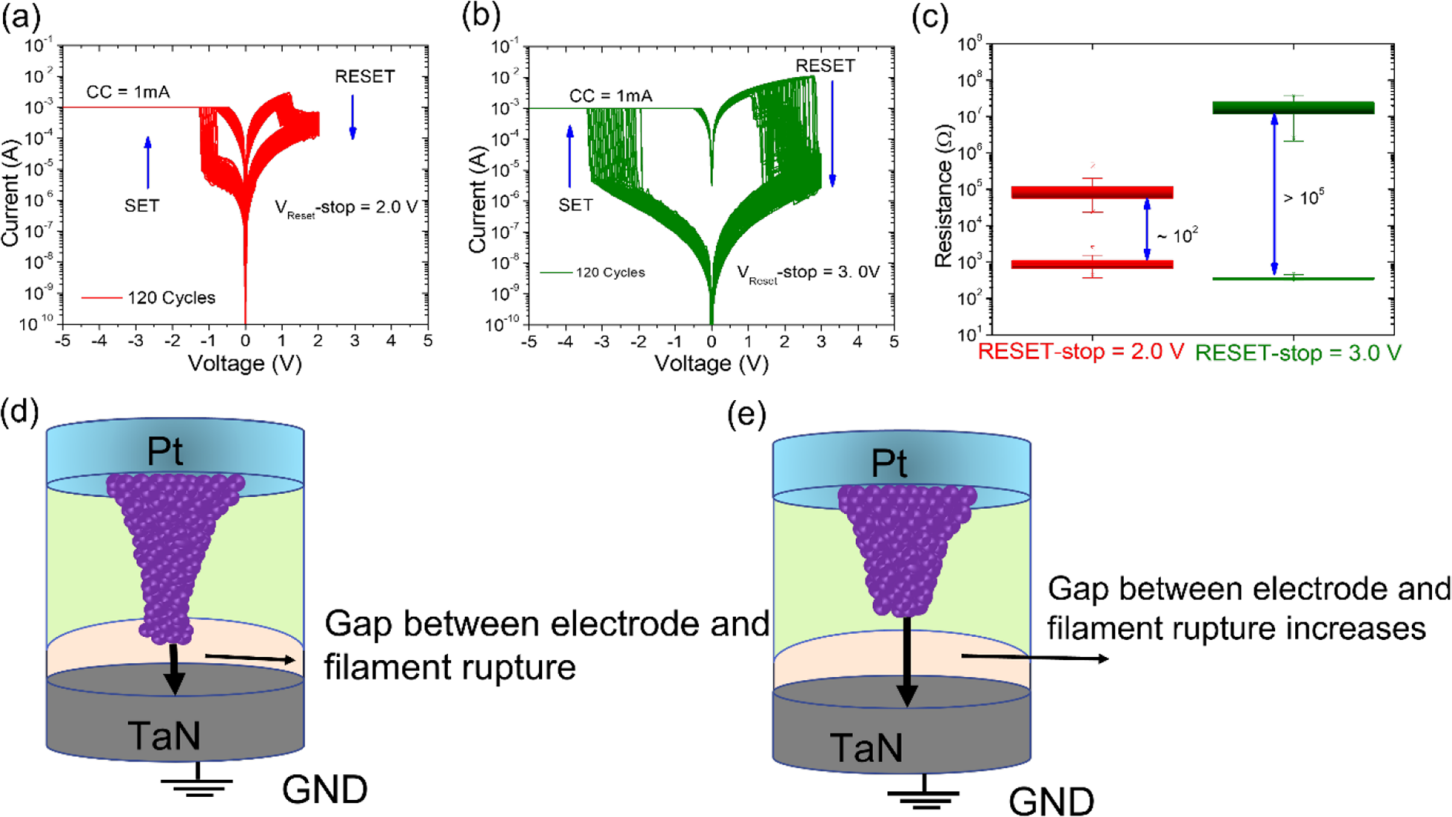
**a** Typical bipolar I–V characteristics and **b** comparison of ON/OFF resistance ratios of the Pt/HfO_2_/Al_2_O_3_/TaN memristive device under different reset-stop voltages, i.e., + 2.0 V and + 3.0 V, with fixed set voltage of − 5 V. **c**, **d** Schematic illustration of switching mechanism of OFF state at different reset-stop voltages (2.0 V and 3.0 V) for the prepared Pt/HfO_2_/Al_2_O_3_/TaN memristive device

The I–V curves of Pt/HfO_2_/Al_2_O_3_/TaN memristive device are drawn in Fig. 4a under different stop voltages, i.e., + 2.0 V and + 3.0 V. Monitoring the compliance current at 1 mA during the set process performed under different reset-stop voltages, electrical resistance in the OFF state is noticed to be distinct for the same individual memory cell. Further note that memristive device gradually switched from LRS to HRS under reset-stop voltage of 2.0 V, which reveals its great potential for application in neuromorphic computing, as gradual switching is necessary for neuromorphic synapses. Moreover, as the memristive device abruptly switched from LRS to HRS on increasing reset-stop voltage to 3.0 V, revealing that it can be used for data storage. To test the ON/OFF ratio at different stop voltages, the 100 consecutive switching cycles were performed 100 times. At a read voltage of 0.2 V, the ON and OFF states were retrieved. Figure 4c depicts the HRS and LRS resistance distributions. Note that by increasing the reset voltage beyond 2.0 V, the memristive device dramatically raised the resistance up to 4 orders of magnitude. It means that conductive filaments are completely ruptured at 3.0 V reset-stop voltage, which is responsible for high ON/OFF ratio. It is concluded that the higher-stop voltages in the reset process, the higher resistance values of HRS could be achieved under the same compliance current. The multi-level storage can be explained by the oxygen vacancy filaments with a conical shape formed by the set process which were thinned and ruptured gradually by controlling the reset-stop voltage [58]. Based on conductive filament theory, we have proposed a physical model to explain the enlargement of ON/OFF ratio or the large memory window [21, 26, 27]. Figure 4d, e shows the schematic diagram of different reset-stop voltage (+ 2.0 V and + 3.0 V)-dependent switching mechanisms of Pt/HfO_2_/Al_2_O_3_/TaN memristive device. The magnitude of HRS level depends on the proportion of filament that dissociates under a positive electric field. When the reset-stop point is small (+ 2.0 V), a slight amount of filament in the dielectric layer dissolves and, consequently, resistance change was less. When the higher positive voltage is applied (+ 3.0 V), the maximum HRS level was achieved. Clearly, a larger tunneling gap between the filament tip and the electrode increases the Schottky barrier height [59]. Because of the expanding Schottky barrier height, the reset current should decrease as the reset-stop voltage rises. The reset current level approaches a steady plateau with only minor fluctuations as the reset-stop voltage is increased. The space between the tip of the conductive filaments and the electrode, which can vary greatly from cycle to cycle, is smaller at lower reset-stop voltage [60]. Besides, by employing greater reset-stop voltage, all conductive filaments were ruptured, which is responsible for decreasing the OFF-state resistance. It means that rupture of the conductive filament is maximum at reset stop of 3.0 V for maximum enlarging the gap between the ruptured filaments, resulting in a higher resistance state. Results show that Pt/HfO_2_/Al_2_O_3_/TaN memristor device has great potential for application in neuromorphic and long data storage.

### Quantized Conductance States

**Fig. 5:**
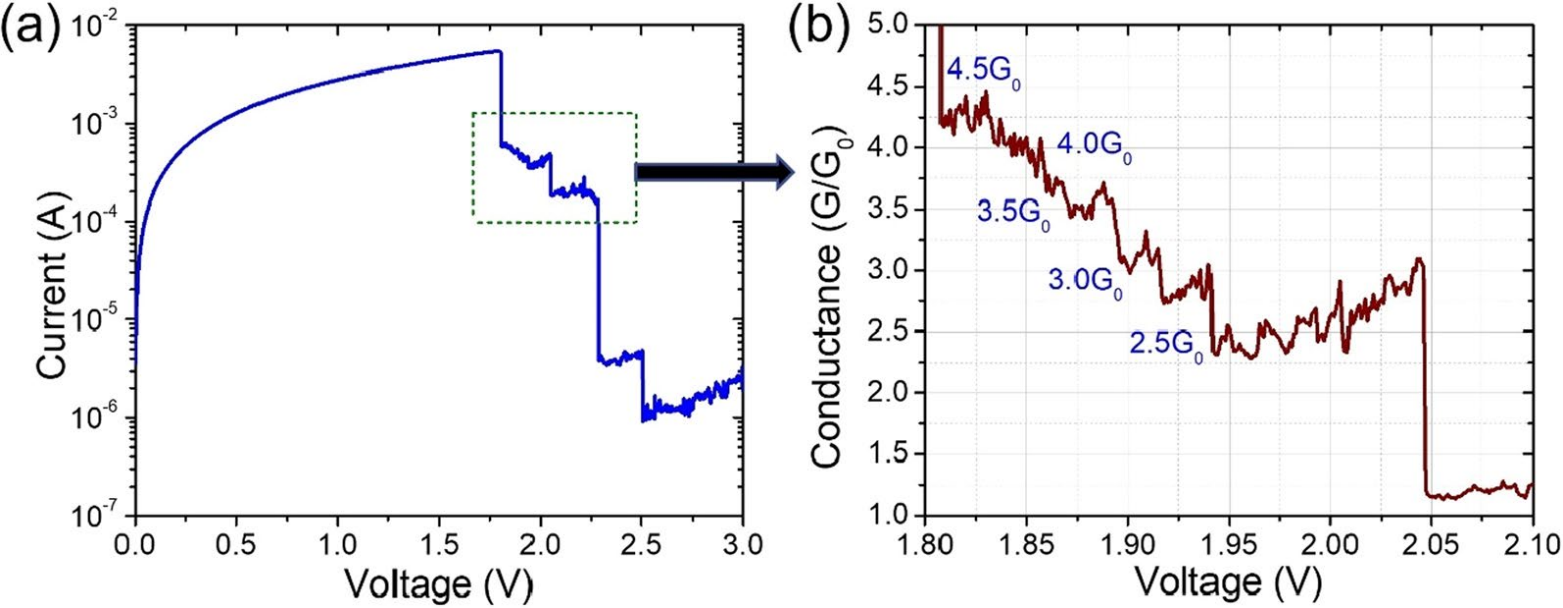
Noticeable quantized changes in the conductance of Pt/HfO_2_/Al_2_O_3_/TaN memristive device: **a** I–V curves during the reset process for slow DC sweep cycles. **b** Conductance–voltage curves revealing the quantum conductance effect. The voltage is increased in steps of 0.005 V and delay time of 0.1 s per step

The quantized effect observed during the reset process is demonstrated in Fig. 5a, which exhibits I–V curves for the slow sweep measurement mode (delay time = 0.1 s, step voltage = 0.005 V). During such reset processes, there noticed drops of current magnitudes in several steps. The corresponding conductance–voltage curves for the reset operation are also drawn as shown in Fig. 5b. Again, voltage is increased in 0.005 V increments with a 1 s delay between each step. The *G* = *I*/*V* was used to compute the electrical conductance (*G*) measured in units of the conductance of a single atomic constant. The quantum of conductance (77.5 µs) is: G_0_ = 2e^2^/h, where e is the charge of an electron and h Planck's constant [61]. When memristive conductance falls below 1 G_0_ at + 2.5 V, the atomic/electronic contact is disconnected, and consequently, memristive device switches to its OFF state. Charge tunneling between the filament and the electrode dominates the conductivity of the memristive device in its OFF state. The charge tunneling distance is continuously extended when positive voltage bias is increased, resulting in a continuous rise in resistance in the OFF state. Multiple conductance states observed in the resistive devices are useful in artificial synapse applications, according to these findings [62–65].

### Pulse Endurance Characteristics

**Fig. 6:**
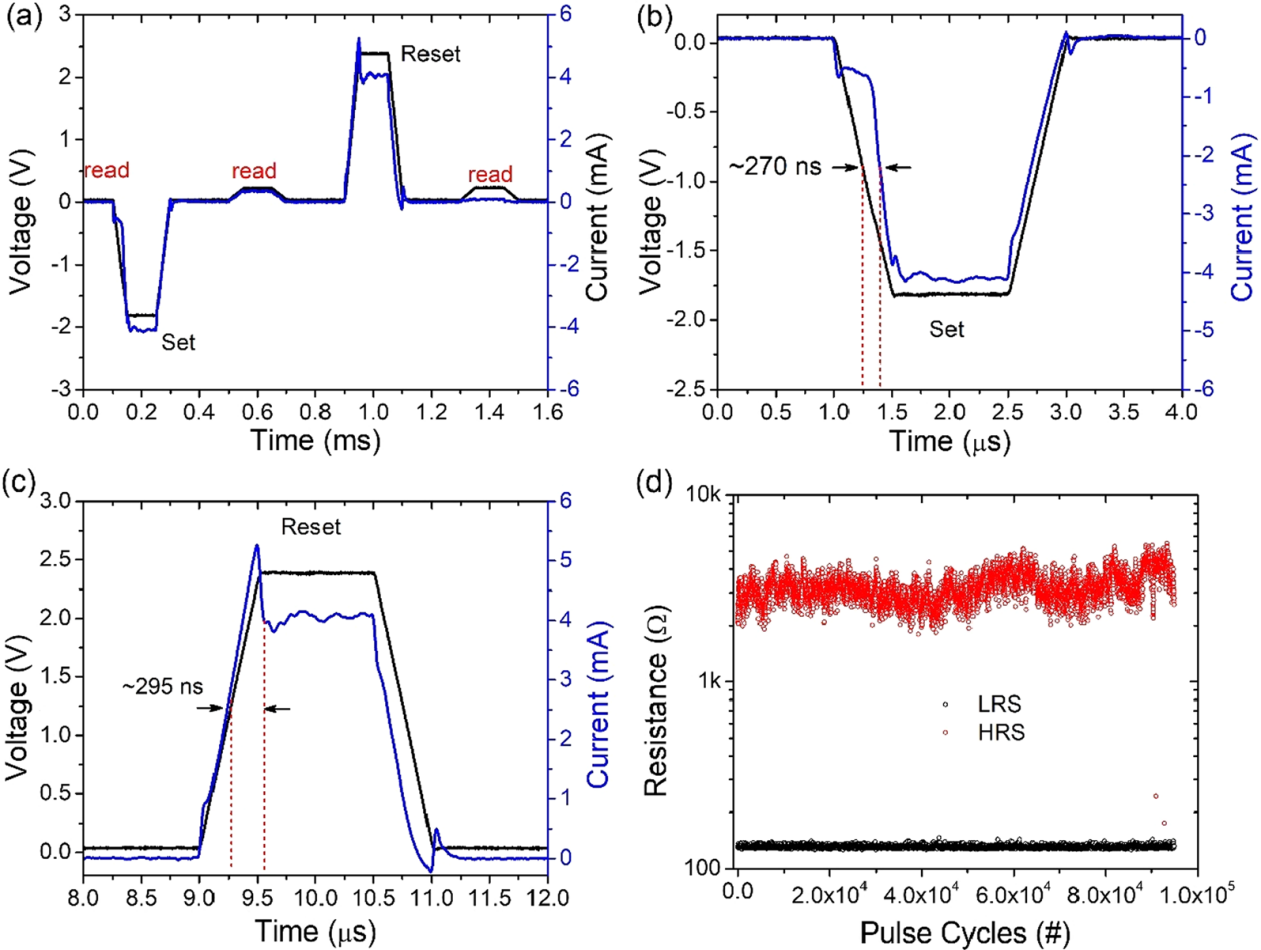
Pulse response characteristics of the Pt/HfO_2_/Al_2_O_3_/TaN memristive device: **a** current response during set and reset process under negative and positive bias pulses, respectively. **b** Set response time is about 270 nm and **c** Reset response time is about 295 ns, which are represented by red dashed lines. **d** Pulse endurance test. Device switched with 10 × difference in HRS and LRS and suffered no read/write disturbance after more than 10^5^ cycles

A pulse mode was initiated to test the switching speed between the ON and the OFF states to further investigate the synaptic characteristics of Pt/HfO_2_/Al_2_O_3_/TaN memristive device. Between the read pulses, we apply either set- or reset-pulse response to demonstrate the short current characteristics. With an effective pulse width of 500 µs, the set/reset voltage was—4.0 V/ + 3.5 V. The typical write/erase speeds of the Pt/HfO_2_/Al_2_O_3_/TaN memristive device under read/set and reset/read bias pulses are shown in Fig. 6, where blue line represents the input bias, and red line represents Pt/HfO_2_/Al_2_O_3_/TaN memristive device response current. As illustrated in Fig. 6a, the read operation (at 0.2 V) before applying the set or the reset pulses clarifies the previous (or initial) state of the device resistance, whereas the read operation after applying the set/reset pulses proves the correctness of the set/reset operation. The delay time between the response current and the input set or the output reset bias in the middle of leading edge is specified as the set/reset speed (Δ*t*). As a result, under the bias of − 2.0 V/+ 2.5, V with 12 µs pulse width, the set and the reset response time of Pt/HfO_2_/Al_2_O_5_/Pt memristive device is about ~ 270 ns and 295 ns, respectively, as illustrated in Fig. 6b, c. As shown in Fig. 6d, continuous alternating current (AC) pulse operation for up to 10^5^ cycles was carried out without failure, proving the high capability for continuous AC operation of this Pt/HfO_2_/Al_2_O_3_/TaN memristive device.

### Neuromorphic Characteristics

**Fig. 7:**
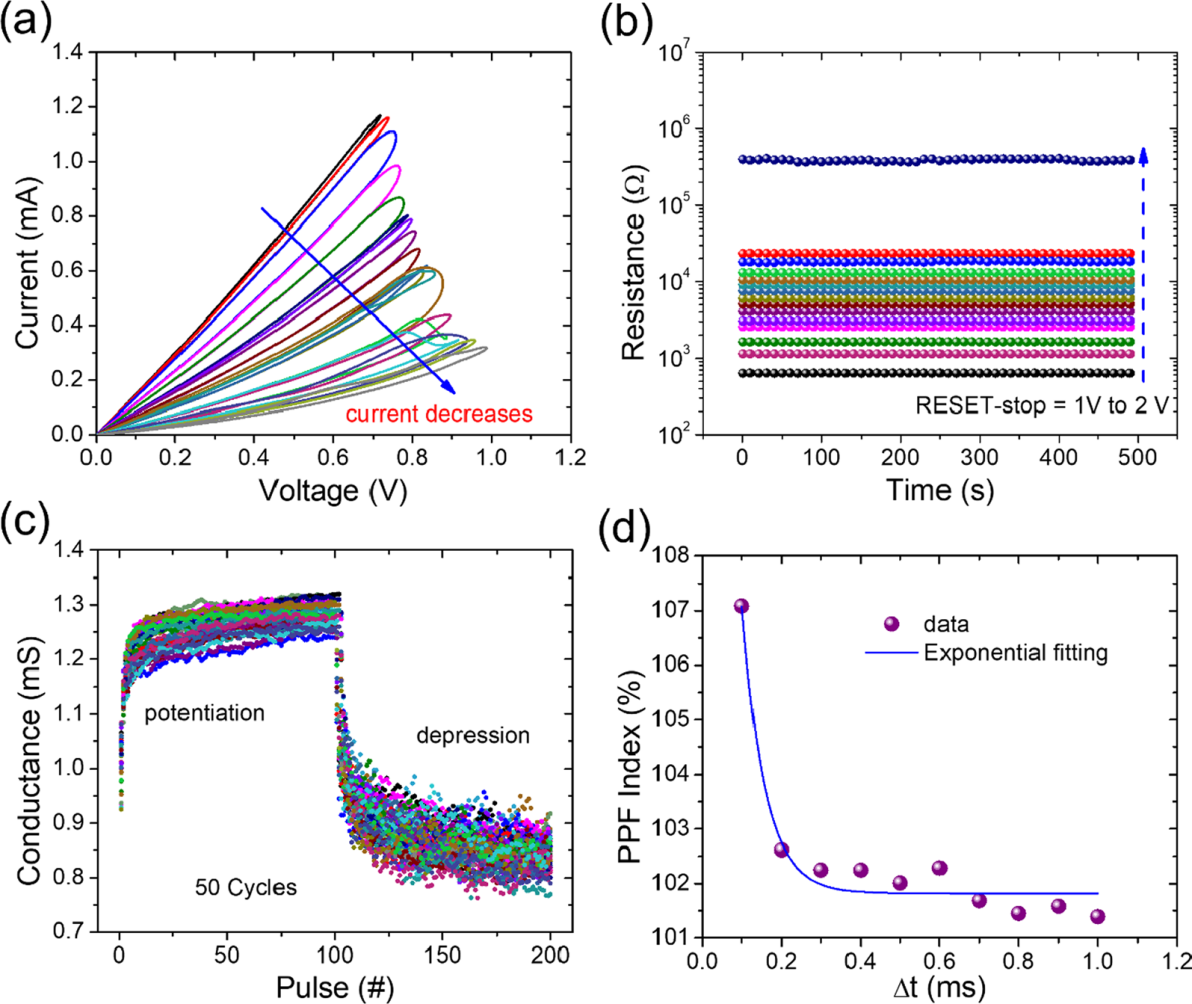
**a** Multilevel conductance states during reset process by applying a consecutive increase in reset voltage sweep from 0.7 to 1.0 V. Arrow indicates the sweep direction. The increment of sweep voltage was 0.01 V. **b** Retention tests were performed at fifteen different HRSs for 500 s. The resistance values were extracted at read voltage of 0.2 V. **c** The potentiation and depression characteristics under 50 negative pulses (− 1.0 V, 500 μs) and 50 positive pulses (+ 1.5 V, 500 μs), respectively. **d** The PPF index vs. pulse interval ∆t, where a series of double spikes with different inter-spike timings were employed, showing an exponential decreasing trend

Multilevel storage is not only a powerful technique to overcome the restrictions of low-density storage, but it also serves as a link between memory and neuromorphic computing. According to the literature, memristive device multilayer conductance capability is an additional property that is beneficial for artificial synapse applications [66–69]. Such multilayer I–V characteristics are shown in Fig. 7a for different reset-stop voltages ranging from 0.7 V to 1.0 V. The 0.01 V was the step increase during this operation. Because of such low increment in the stopping voltage, fifteen conductance states were obtained that correspond to partial rupturing of oxygen vacancies-based conductive filaments [70]. Multilevel retention tests were also carried out by varying the reset-stop voltage (0.7 V to 2.0 V), as shown in Fig. 7b. During continuous stress of 0.2 V for 500 s, retention tests of fifteen different states were performed, which verify the nonvolatile behavior and indicate that the memristive device is suitable for multi-level data storage memory. Additionally, potentiation and depression tests were performed for 50 consecutive cycles to imitate the synaptic function of the memristive device, as illustrated in Fig. 7c. Conductance of the memristive device can effectively be potentiated (conductance gradually increases) and depressed (conductance gradually lowers) using fifty negative pulses and fifty positive pulses, respectively. To activate the potentiation and depression features in the resistive device, the pulse amplitude was tuned to − 1.0 V/+ 1.5 V, while the pulse width to 500 s. Notice that after 40 cycles of potentiation and depression, the memristive device remained intact leading to its strong stability and repeatability so that it can be utilized to build artificial synapses in neuromorphic computing systems. The PPF (paired-pulse facilitation) is a physiological phenomenon linked to short-term plasticity in which a previous stimulus enhances a synaptic response to a future stimulus [71]. The interval between the two pulses was altered from very short to extremely long to assess the memristive device's PPF behavior over a wide variety of pulse intervals. Figure 7d illustrates such measured PPF behavior of the memristive device at various pulse intervals. As analogy with biological synapses, PPF index drops as the pulse interval increases [72]. Calculating a PPF index from the expression (I_2_/I_1_), where I_1_ and I_2_ are the peak amplitudes of the first and second responses, respectively, can be used to estimate changes in the PPF [73]. The PPF index for double pulse stimulation with the smallest pulse interval was the highest (i.e., 107). The dual stimuli with a longer gap, on the other hand, had the lowest PPF index of 102. PPF behavior was absent or inadequate in double stimulations with much longer pulse intervals. The PPF index in biological synapses is generally dependent on the interval between paired presynaptic stimulations, which our memristive device successfully simulates.

### Resistive Switching Mechanism

**Fig. 8:**
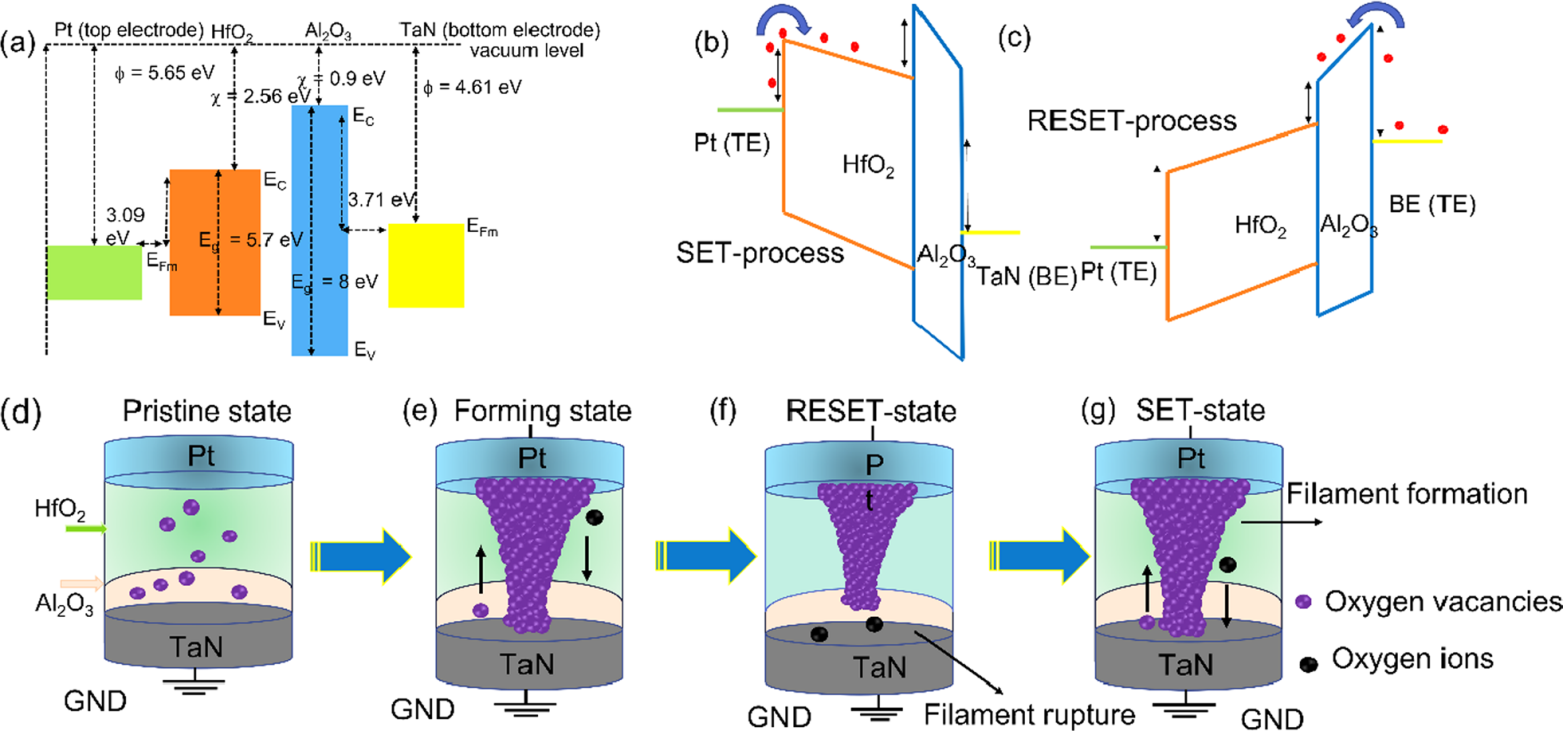
Relative energy band diagrams and conduction model: **a** before applying electric field (zero bias), **b** ON state (forward bias), and **c** OFF state (reverse bias) of Pt/HfO_2_/Al_2_O_3_/TaN memristive device. Physical model of resistive switching behaviors: **d** pristine state, **e** set process (conductive filaments creation under negative bias), and **f** reset process (filament ruptured under positive bias) of the Pt/HfO_2_/Al_2_O_3_/TaN memristive devices

We propose a model to provide comprehensive insight into the RS mechanism of the pristine state, set state, and reset state of the Pt/HfO_2_/Al_2_O_3_/TaN memristive device, based on the above-mentioned electrical and other material characteristics research, as shown in Fig. 8. The memristive device switching behavior and performance are determined by the work function (*ϕ*) difference between the electrodes (top and bottom), as well as the electron affinity (*χ*) difference between the switching layers (HfO_2_ and Al_2_O_3_). TaN has a value of work function (*ϕ*) 3.4 eV, which is lower than Pt (5.65 eV). [74] The *χ* values of HfO_2_ and Al_2_O_3_ are 2.85 eV and 0.9 eV, respectively [75, 76]. *E*_v_ and *E*_c_ represent the valence and conduction band edges of HfO_2_, respectively, while *E*_g_ represents the band gaps, which are 5.8 eV (HfO_2_) and 8.0 eV (Al_2_O_3_). The Al_2_O_3_ has much lower value of *χ* but possesses higher band gap value than that of HfO_2_, allowing electrons to move from Al_2_O_3_ to HfO_2_ layer (possessing higher electron affinity), resulting in larger electron concentration in the dielectric matrix. The differences between the values of *ϕ* and *χ* are responsible to create a barrier height. As a result, the junction produced by the TaN bottom electrode and the Al_2_O_3_ interlayer has a substantial Schottky barrier of around 3.71 eV, which is higher than the 3.09 eV for the interface formed by the Pt top electrode and the HfO_2_ layer, as shown in Fig. 8. An asymmetric Schottky barrier is projected to emerge at the top HfO_2_/Pt contact due to the energy difference. Analog bipolar switching is caused by a work function mismatch of 2.25 eV between the inert Pt top electrode (5.65 eV) and the high oxygen affinity TaN bottom electrode (3.4 eV) [77]. Figure 8a shows the steady-state conduction band diagram at zero bias. When a negative voltage bias is supplied to the top Pt electrode, Schottky barrier Al_2_O_3_/TaN bottom interface regulates electron drift toward TaN bottom electrode at a negative voltage, as illustrated in Fig. 8b. Oxygen ions transport toward the Al_2_O_3_ layer and hence to the TaN bottom electrode from the upper HfO_2_ layer. Oxygen ions return to the Al_2_O_3_ and HfO_2_ layer when a positive bias is supplied to the Pt top electrode. As shown in Fig. 8c, the Schottky barrier at the Pt/HfO_2_ interface limits electron injection at positive voltage and is limited by the discontinuity of the conduction band at the Al_2_O_3_/HfO_2_ contact. Based on the XPS results (see Fig. 1), a potential switching mechanism for the Pt/Al_2_O_3_/HfO_2_/TaN memristive is presented, which considers the TaN electrode chemical activity with oxygen and the non-distribution of oxygen vacancy concentration in the HfO_2_/Al_2_O_3_ dialectics.

Finally, a conducting model is proposed to explain the resistance switching mechanism based on the results of electrical measurements. The role of TaO_x_N_y_ in the reset and set process is the key to understanding this proposed combined model. Previously reported that TaN electrode act as a reservoir for oxygen ions drifting under the applied voltage [10]. Because the Gibbs free energies of the interfacial TaO_x_N_y_ layer (− 604.0 kJ/mole) is higher as compared to Al_2_O_3_ layer (-1076.0 kJ/mole) and as well as upper HfO_2_ layer (− 1010.8 kJ/mole).[78, 79]. The set and reset occurs at TaO_x_N_y_ interface which also acts as the switching region. Figure 8d displays the pristine state of the memristor device. When a negative voltage is applied on the Pt top electrode caused oxygen ions to drift from the insulating layer to the high oxygen affinity TaN bottom electrode, leading to the formation of TaO_x_N_y_ interfacial layer. The memristive device switches from OFF state to ON state (forming), as shown in Fig. 8e. When a reverse reset voltage is applied to the memristive device, the bottom electrode TaO_x_N_y_ acts as an oxygen reservoir. The oxygen ions migrate back to the high-k oxide layer and fill the oxygen vacancies. Then, a gap forms in the insulating layer and consequently the tip of conductive filaments is allowing to reset back from ON to OFF state, as shown in Fig. 8f. Moreover, the insulating gap helps to enhance the electric field between the filament and metal electrode, and therefore, oxygen ions in the insulating layer can overcome the barrier from the metal [80]. These oxygen ions can recombine with the vacancy to form the insulating oxide at the filament tip. Therefore, the resistance increases (Fig. 4). Again, by applying negative bias to the top electrode, oxygen ions migrate from high-k insulating layer to TaO_x_N_y_ interfacial layer, forming conductive filaments again between the two electrodes and switching the device from OFF to ON state (Fig. 8g). In this switching model, it is easy to explain that the bipolar switching phenomena can easily occur in Pt/HfO_2_/Al_2_O_3_/TaN structure with a non-symmetrical electrode configuration. Besides as an oxygen reservoir, the TaO_x_N_y_ interfacial layer also functioned as oxygen ion barrier [81, 82] from the resistive switching function layers, which may be responsible for improved switching endurance property, tight distribution in set/reset voltages, extended duration stability, and large memory window of Pt/HfO_2_/Al_2_O_3_/TaN structure.

Results of performance comparison between current work and previously reported HfO_2_-based bilayer and trilayer memristors are summarized in Table 1. Compared to other bilayer and trilayer memristors, HfO_2_-based bilayer resistive switching memristor demonstrated noticeable advantages such as lower operating voltages, high endurance, and large ON/OFF ratio (> 10^5^). These comparative analyses signify that bilayer HfO_2_-based memristor is potential candidates for nonvolatile memory and neuromorphic systems.

**Table 1:** Performance comparison of HfO_2_-based bilayer and trilayer memristor

Memristor structure	Forming voltage (V)	Operating voltage (V)	ON/OFF ratio	Endurance (cycles)	Retention (s)	Switching polarity	Switching mechanism	References
Pt/HfO_2_/Al_2_O_3_/TaN	− 5.06	− 2.7/+ 1.9	~ 10^5^	1000	10^4^ s	Bipolar	ECM	This work
Ti/TiO_2_/HfO_2_/Si	Free	+ 10/− 6.0	~ 10^2^	~	10^4^ s	Bipolar	VCM	[83]
Al/AlO_x_/HfO_x_/Pt	~	+ 1/− 2.0	~ 103	~ 400	10^4^ s	Bipolar	VCM	[84]
Pt/HfO_2_/SiO_2_/TaN	− 5.0	− 1.9/+ 2.7	~ 10^4^	1000	10^4^ s	Bipolar	ECM	[52]
Pt/Al_2_O_3_/HfO_2_/HAlfO_x_/TiN	Free	− 1.0/+ 1.2	~ 10^2^	1000	10^4^ s	Bipolar	ECM	[11]
TaN/HfO_2_/Al2O_3_/HfO_2_/ITO	–8.2	− 0.8/+ 1.0	~ 10	1000	10^4^ s	Bipolar	VCM	[85]

## Conclusions

By inserting the high band gap and low Gibbs free energy thin Al_2_O_3_ interlayer, high resistive switching stability, neuromorphic synapses, and quantum conductance were demonstrated in the Pt/HfO_2_/Al_2_O_3_/TaN memristive device. HRTEM study confirms the structure and thicknesses of the high-k dielectric HfO_2_ and Al_2_O_3_ films. A small layer of TaO_x_N_y_ occurs at the Al_2_O_3_/TaN contact because of Ta strong oxygen accumulation characteristic, as shown by XPS analysis. The Pt/HfO_2_/Al_2_O_3_/TaN memristive device demonstrates stable analog bipolar resistive switching behavior with five orders of ON/OFF resistance ratio, according to the results. For retention and endurance tests, the above-indicated ON/OFF resistances ratio in the LRS and HRS was maintained after continuous 1000 DC switching cycles, 10^5^ AC switching cycles, and nonstop ± 0.2 V stress up to 10^4^ s. Quantum conductance was also found in the reset process when the DC voltage sweep rate was reduced. Furthermore, for high-density storage, multilayer conductance states were controlled by interrupting reset voltage. During the set and reset processes, the switching speed was to be 270 ns and 295 ns, respectively. Finally, biological synaptic actions like long-term potentiation (increases), long-term depression (decreases), and paired-pulse facilitation timing are all rigorously proven. This research demonstrates that using high band gap and Gibbs free energy thin layer of Al_2_O_3_ as an interlayer can improve ON/OFF ratio, uniformity, stability, and reproducibility. The memristive device also has significant promise in multilevel quantum conductance and artificial synapses applications, although current memristive device still has disadvantages such as high-power consumption, abrupt set/reset switching, and electroforming process. In future work, we will try to overcome these issues by interface engineering of the memristive devices.

## Supplementary Information

**Additional file 1**. Supplementary Materials .

## Data Availability

All data generated or analyzed during this study are included in this article and its supplementary information file.

## Abbreviations

LRSs and HRSs: Low- and high-resistance states
PPF: Paired-pulse facilitation
ALD: Atomic laser deposition
TMA: Trimethylaluminum
TDMAHF: Tetrakis dimethylamino hafnium
HRTEM: High-resolution transmission electron microscope
XPS: X-ray photoelectron “HRS and LRS of > 10^5^” spectroscopy
IRS: Initial resistance state
DC: Direct current
Δ*t*: Speed
*ϕ*: Work function
*χ*: Electron affinity
AC: Alternating current
ECM: Electrochemical metallization
VCM: Valence change mechanism
