# Supplementary material for: Robust Resistive Switching Constancy and Quantum Conductance in High-k Dielectric-Based Memristor for Neuromorphic Engineering
Source: Nanoscale Res Lett. 2022 Jun 24;17:61. doi: 10.1186/s11671-022-03699-z (PMC9232664; doi:10.1186/s11671-022-03699-z)
Supplement: Supplementary file 1 — Additional file 1. Supplementary Materials . [file 11671_2022_3699_MOESM1_ESM.docx]

Supplementary Materials

Robust Resistive Switching Constancy and Quantum Conductance in High-k Dielectric Based Memristor for Neuromorphic Engineering

Muhammad Ismail^a^, Chandreswar Mahata^a^, Myounggon Kang^b^*, Sungjun Kim^a*^

^a^Division of Electronics and Electrical Engineering, Dongguk University, Seoul 04620, Republic of Korea

^b^Department of Electronics Engineering, Korea National University of Transportation, Chungju-si 27469, Republic of Korea

^*^Corresponding authors: [mgkang@ut.ac.kr](mailto:mgkang@ut.ac.kr) (M. Kang) and [sungjun@dongguk.edu](mailto:sungjun@dongguk.edu) (S. Kim)


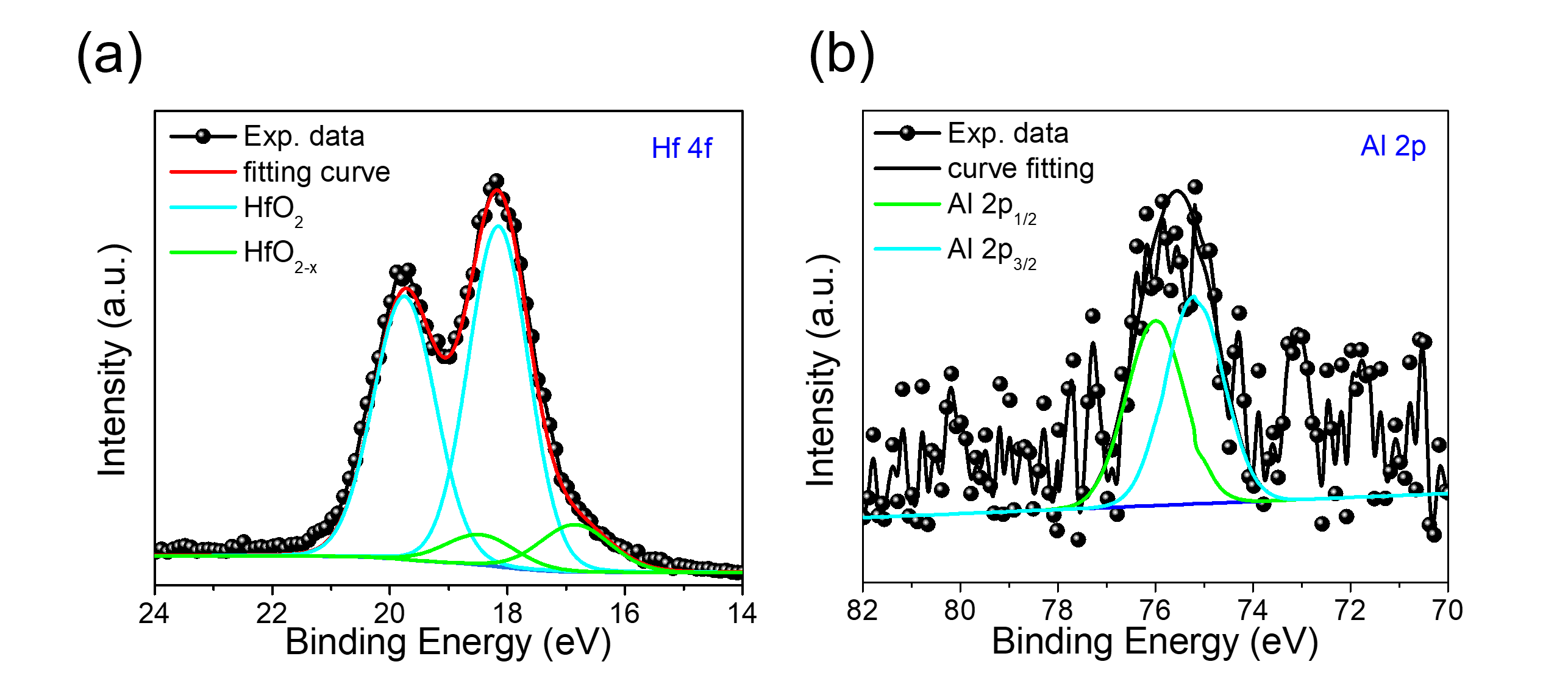


Fig. S1. XPS spectra of (a) Hf 4f, and Al2 2p, respectively.


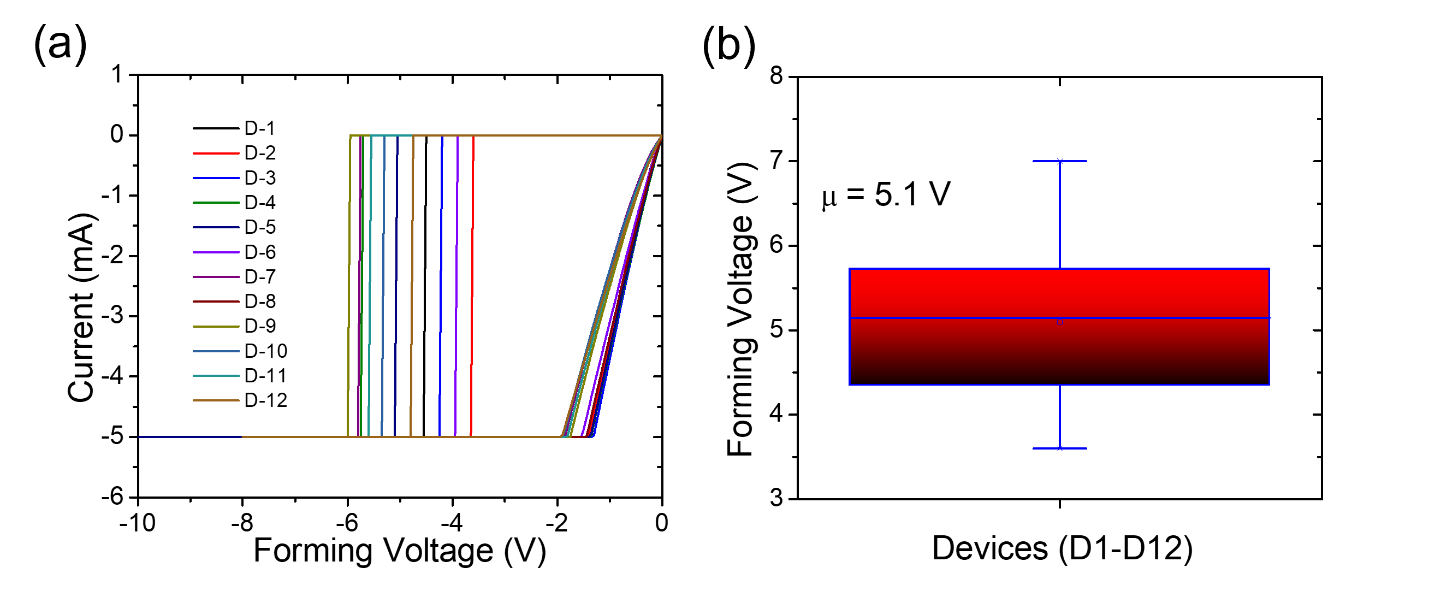


Fig. S2. (a) Typical electroforming I-V curves, and (b) statistical distribution of the electroforming voltage of the twelve randomly selected memristive cells.


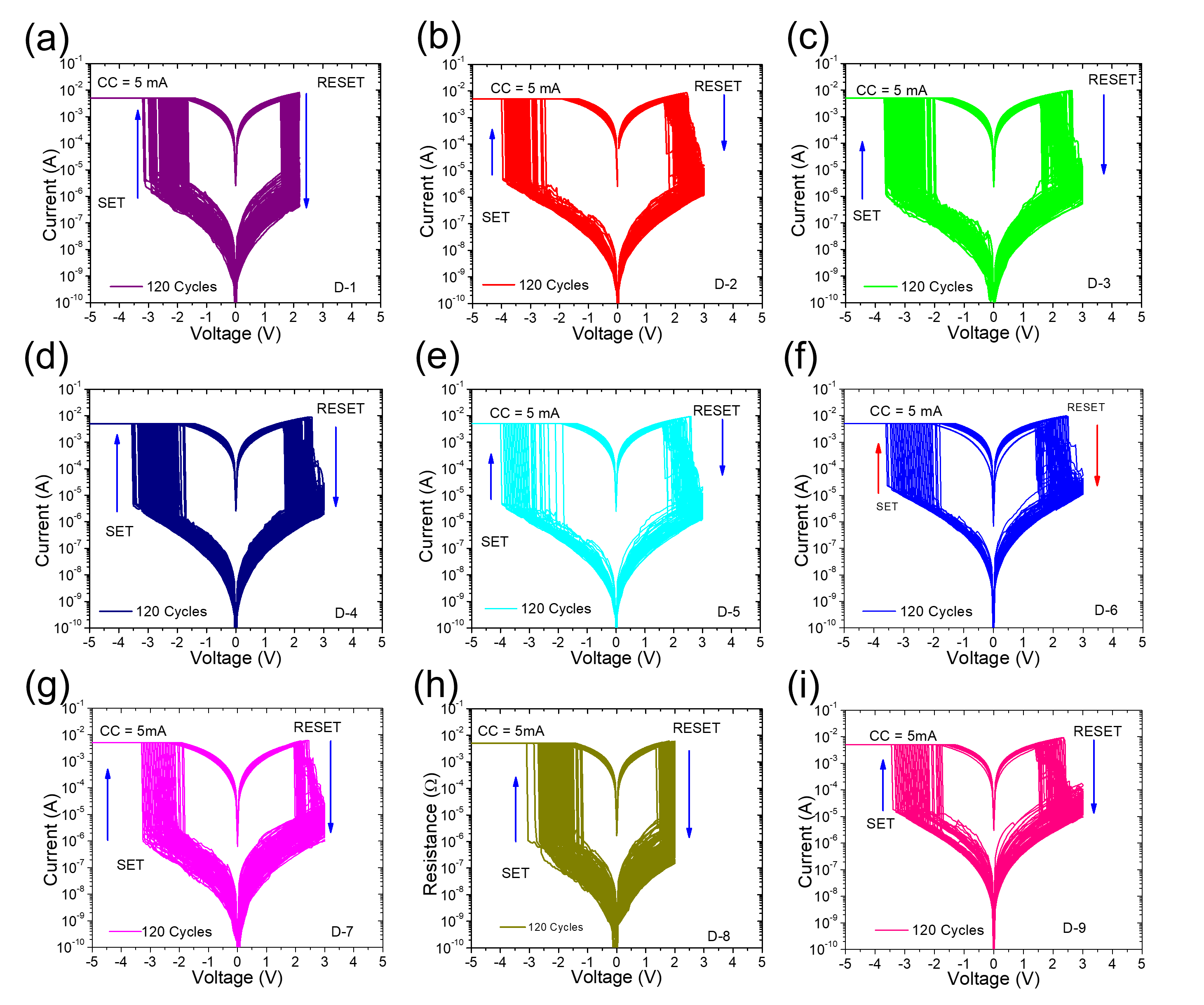


Fig. S3. (a-i) Device to device resistive switching performance of the Pt/HfO_2_/SiO_2_/TaN memristive device, where 120 consecutive cycle-to-cycle sweep were repeated.


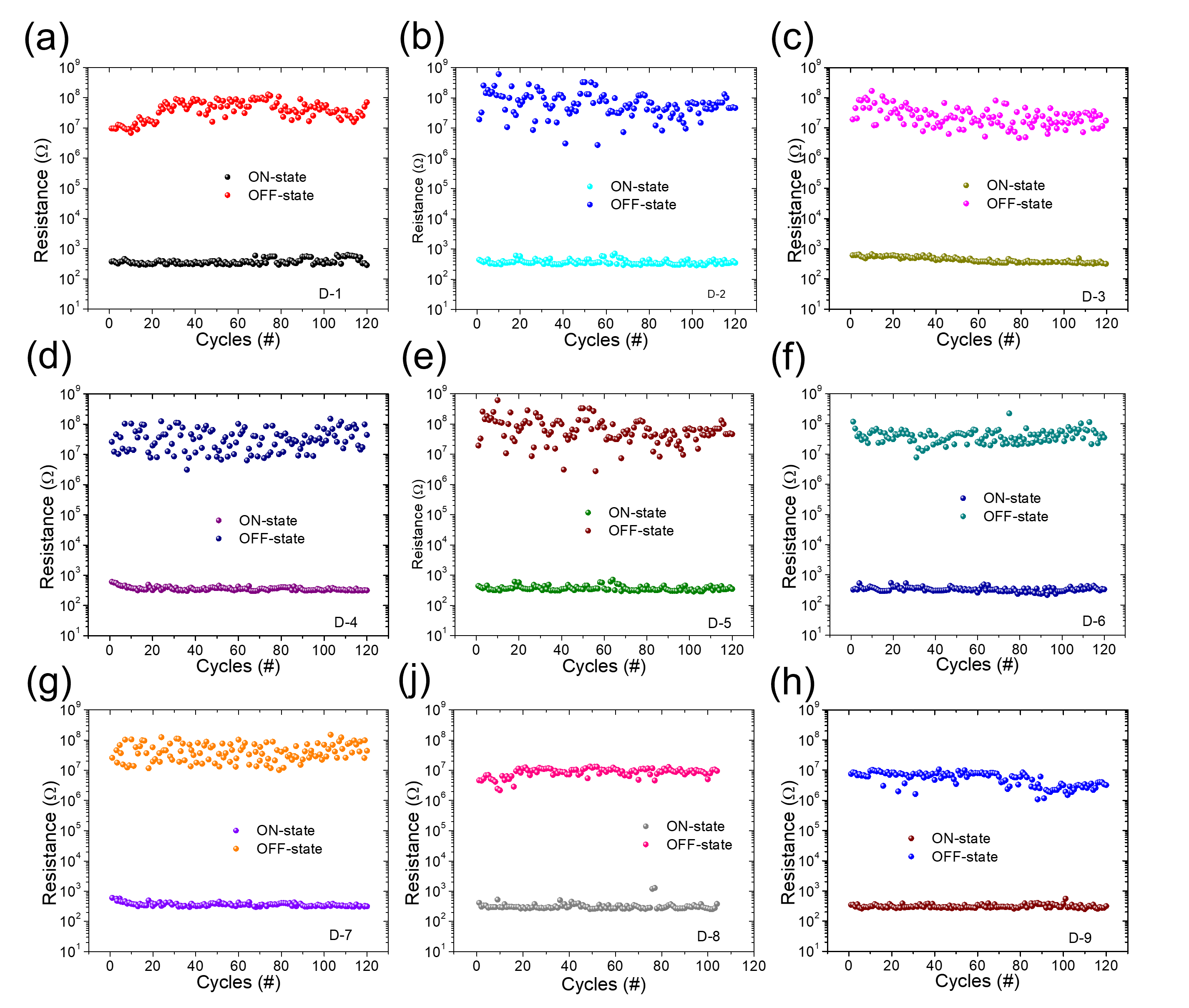


Fig. S4. (a-i) Device to device endurance switching performance of the Pt/HfO_2_/SiO_2_/TaN memristive device. The ON- and OFF-state were measured at read voltage of 0.2 V.


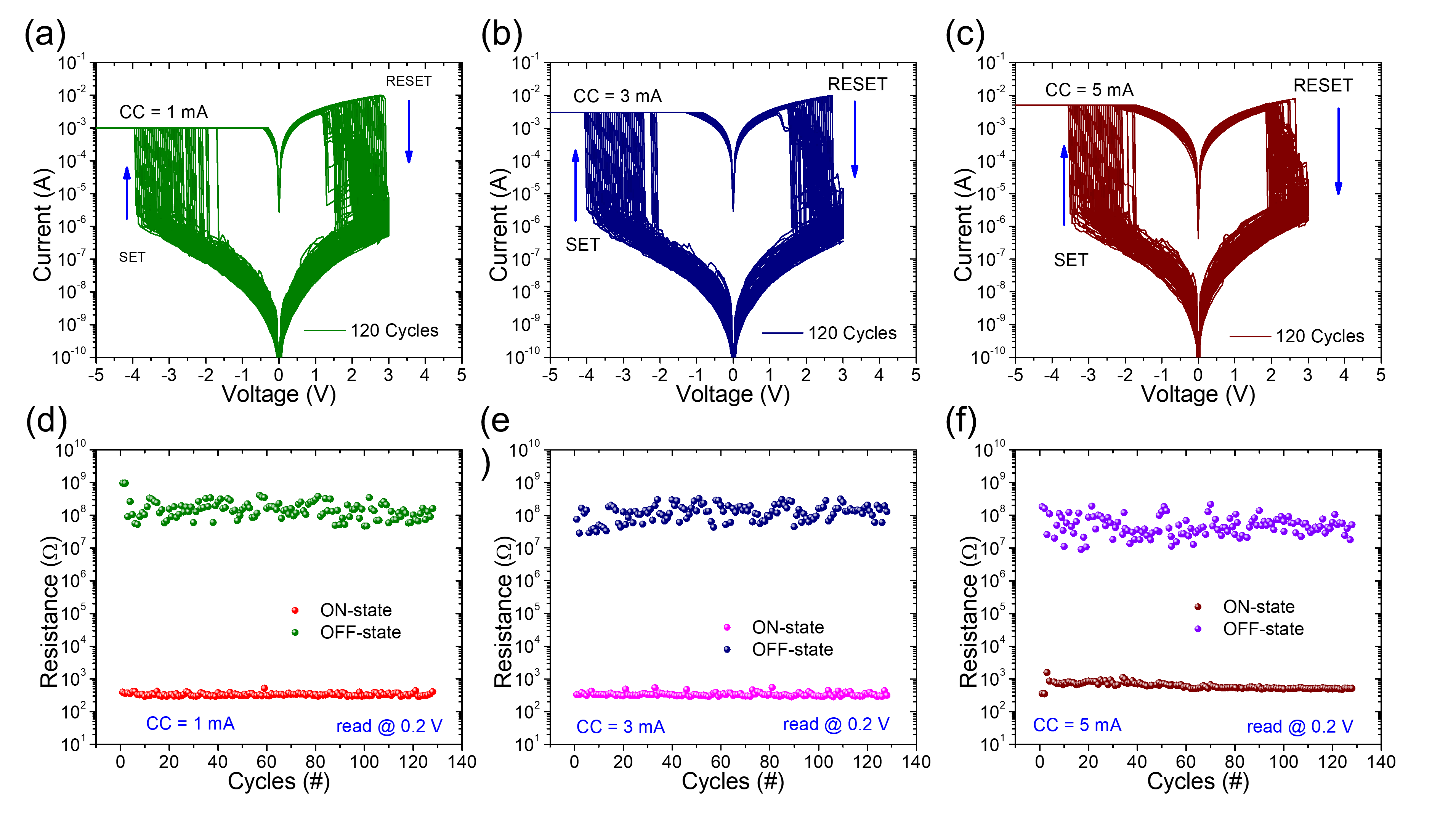


Fig. S5. (a-c) I-V curves, and (d-f) endurance performance of the Pt/HfO_2_/SiO_2_/TaN memristive device under different compliance currents.


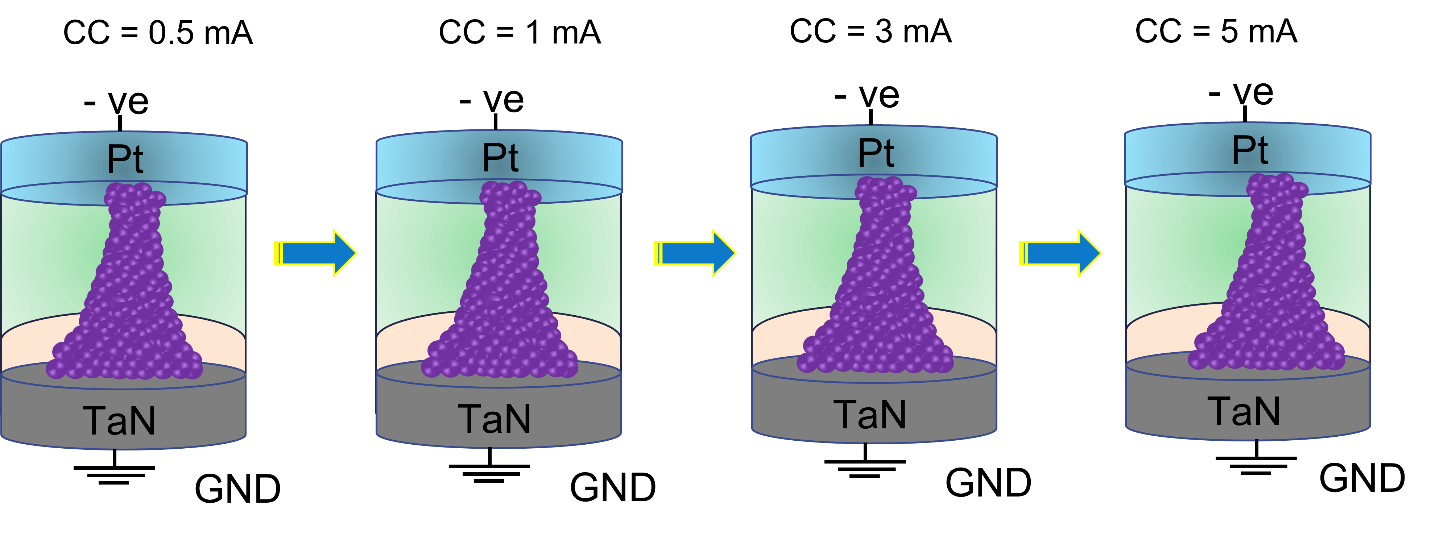


Fig. S6. Schematic illustration of switching mechanism of multilevel switching characteristics under different current compliance. The size of conductive filaments does not change even by accumulation of more oxygen vacancies under different current compliance of the Pt/HfO_2_/SiO_2_/TaN memristive device.
